# Supplementary material for: Photodegradation of Riboflavin under Alkaline Conditions: What Can Gas-Phase Photolysis Tell Us about What Happens in Solution?
Source: Molecules. 2021 Oct 3;26(19):6009. doi: 10.3390/molecules26196009 (PMC8512791; doi:10.3390/molecules26196009)
Supplement: Supplementary file 1 [file molecules-26-06009-s001.zip › molecules-1358311-supplementary.pdf]

Supplementary Materials

Photodegradation of Riboflavin under Alkaline Conditions: What can Gas-Phase Photolysis Tell Us About What Happens in Solution?

Natalie G. K. Wong,[a] Chris Rhodes,[a] and Caroline E. H. Dessent[a]*

[a] Department of Chemistry, University of York, Heslington, York, YO10 5DD, U.K.

* Corresponding author: Email: caroline.dessent@york.ac.uk

Supporting Information:

S1. Photodepletion laser power dependence measurements

S2. Electron detachment yield *vs*. photodepletion yield interpretation

S3. Higher-energy collisional dissociation of [RF–H]$^{-}$

S4. Solution-phase photofragment production curves

S5. Design of custom-made on-line syringe cell

S6. Control solution-phase studies

S1. Photodepletion laser power dependence measurements

Laser power measurements were conducted on $[RF{-}H]^-$ at several of its respective absorption maxima when electrosprayed in deionized H_2O. The plot displayed in Figure S1 shows the parent ion photodepletion intensities (Int_{OFF} – Int_{ON}) at the selected photon energies. Following standard protocols, the data has been plotted and fit to a power function.[1–3] The resultant slope is thereby proportional to the number of absorbed photons implicated in the experiment(s).

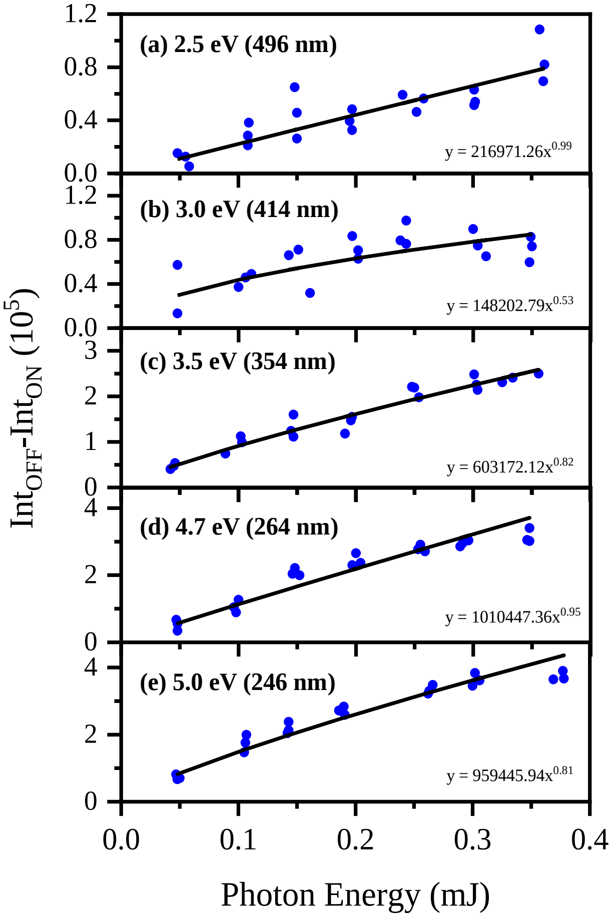

Figure S1. Power dependence measurements for $[RF{-}H]^-$ at five absorption maxima of **(a)** 2.5 eV (496 nm), **(b)** 3.0 eV (414 nm), **(c)** 3.5 eV (354 nm), **(d)** 4.7 eV (264 nm), and **(e)** 5.0 eV (246 nm).

Multiphoton events *via* instantaneous absorption of multiple photons in the Franck-Condon region are negligible as the laser beam is only softly focused through the ion-trap region. The slopes of all photon energies are less than 1.0, confirming that photodepletion $[RF{-}H]^{-}$ at 0.1 mJ are evidently not multiphoton in nature.

S2. Electron detachment yield *vs*. photodepletion yield interpretation

In Figure S2 where we present ED* spectra, we overlay such data with the photodepletion yield (PD*) for ease of comparison; PD* is the normalized photodepletion ion count (Eq. 6), which provides the most straight-forward comparison to ED* (Eq. 5). Previous work on the vertical detachment energies (VDEs) of flavins have shown predicted VDEs of *ca.* 4.0 and 3.8 eV for deprotonated alloxazine and *ca.* 4.6 and 4.7 eV for deprotonated structures of lumichrome [4] with deprotonation on the ribose side chain likely giving a VDE close to that of an alkoxide [5]. While electron detachment is observed here for $[RF–H]^{–}$ from around 3.3 eV, it may be that indirect electron detachment below the VDE is occurring for this molecule following excitation of an electronic transition.

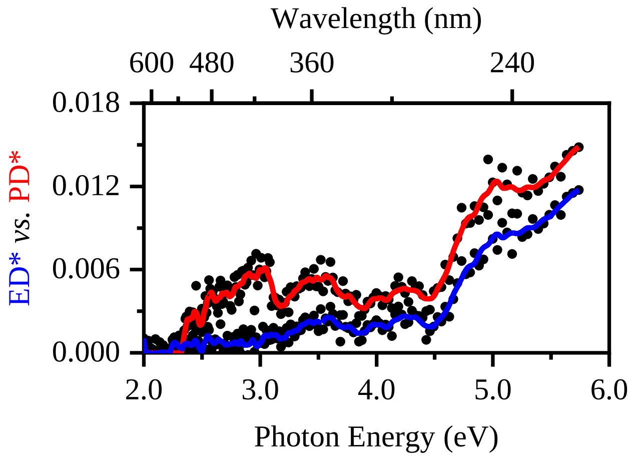

Figure S2. (a) Electron detachment yield (ED*; blue) *vs*. photodepletion yield (PD*; red) of $[RF–H]^{–}$. The solid lines are a five-point adjacent average of the data points.

S3. Higher-energy collisional dissociation of [RF–H]$^-$

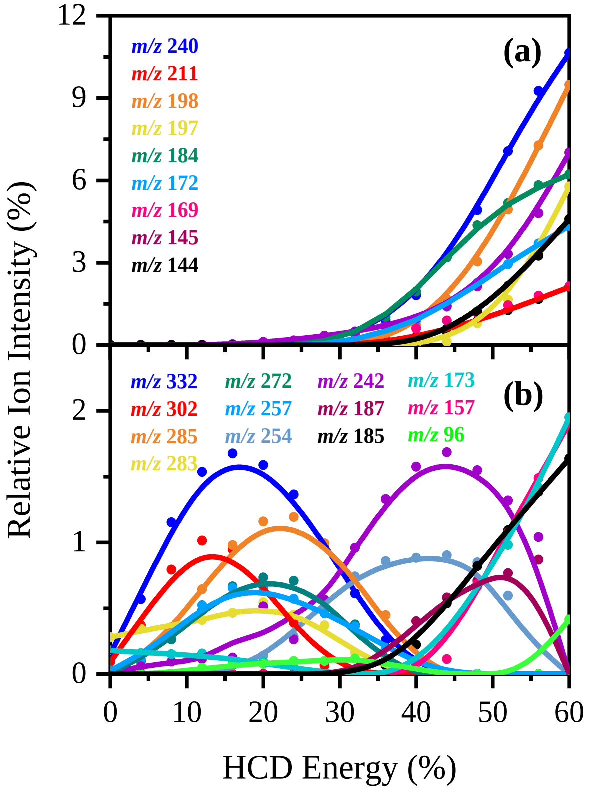

Figure S3. Parent ion dissociation curves [RF–H]$^-$ (*m/z* 375) and the extent of the minor thermal fragments produced between 0% and 60% HCD energy, as electrosprayed in EtOH. The curved lines are a five-point adjacent average of such data points and are provided as a viewing guide, to emphasize the profile for each individual fragment.

S4. Solution-phase photofragment production curves

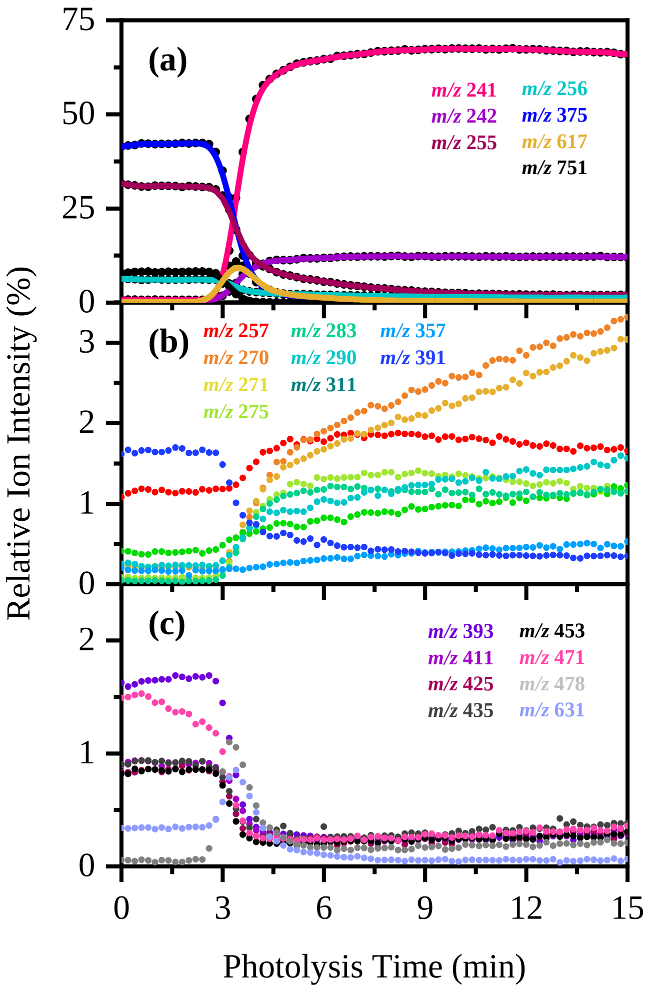

Figure S4. (a) Relative ion intensity plot highlighting the solution-phase photofragment ions of aqueous RF produced over a 15 minute interval of irradiation at 365 nm when delivered *via* ESI-MS in the negative ion mode. **(b-c)** Linked inset plot highlighting the relative ion intensities of the minor photofragments observed upon the solution-phase irradiation of aqueous RF. The curved lines included with the data points are a three-point adjacent average of such data points and are extracted from an average of 3 repeat runs.

S5. Design of custom-made on-line syringe cell

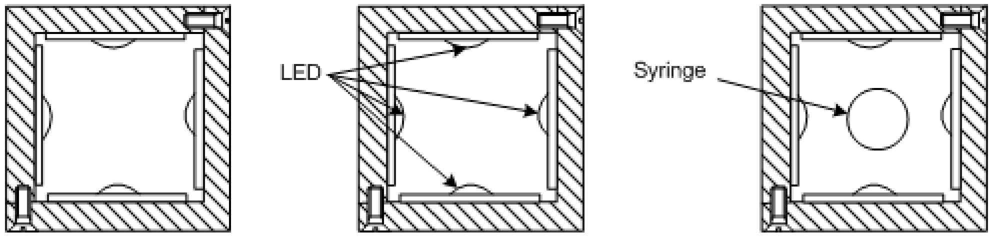

Figure S5. Various cross-sectional views of the custom-made on-line syringe photolysis cell.

S6. Control solution-phase studies

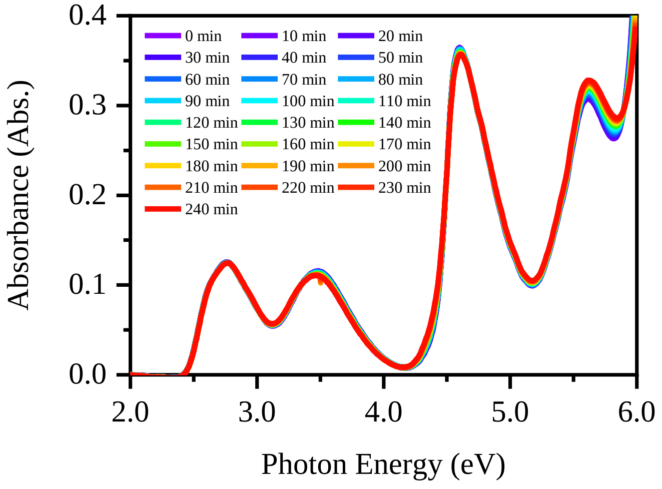

Figure S6. Static UV-visible absorption spectra monitoring the absorption spectrum of riboflavin, prepared in aqueous solution with trace amounts of NH_3 (0.4% v/v), between 0–240 min. The overall stability of the spectra depicted support the idea that hydrolysis does not occur on this time scale.

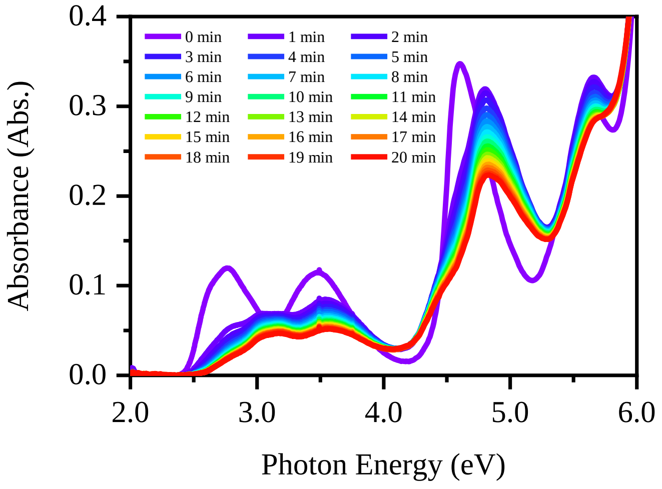

Figure S7. Static UV-visible absorption spectra monitoring the changes in the absorption spectrum of riboflavin, prepared in aqueous solution with trace amounts of NH_3 (0.4% v/v), between 0–20 min upon irradiation (365 nm; 50% light int.).

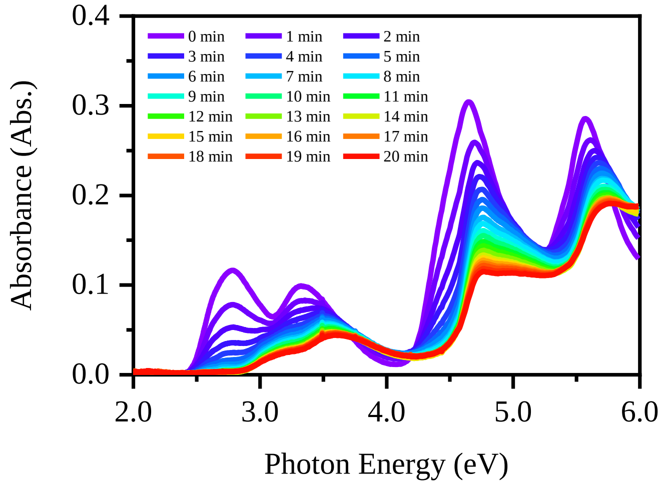

Figure S8. Static UV-visible absorption spectra monitoring the changes in the absorption spectrum of riboflavin, prepared in aqueous solution, between 0–20 min upon irradiation (365 nm; 50% light int.).

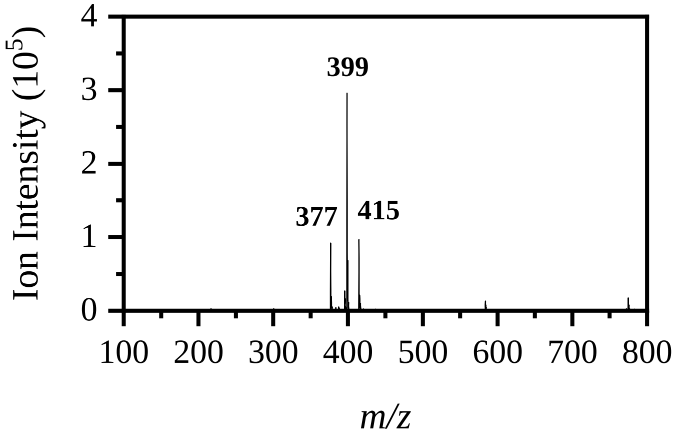

Figure S9. Positive ion mode electrospray ionization (ESI) mass spectrum of protonated riboflavin ([RF+H]$^+$) at m/z 377. Note that the ions at noted at *m/z* 399 and 415 represent its [RF+Na]$^+$ and [RF+K]$^+$ systems, respectively.

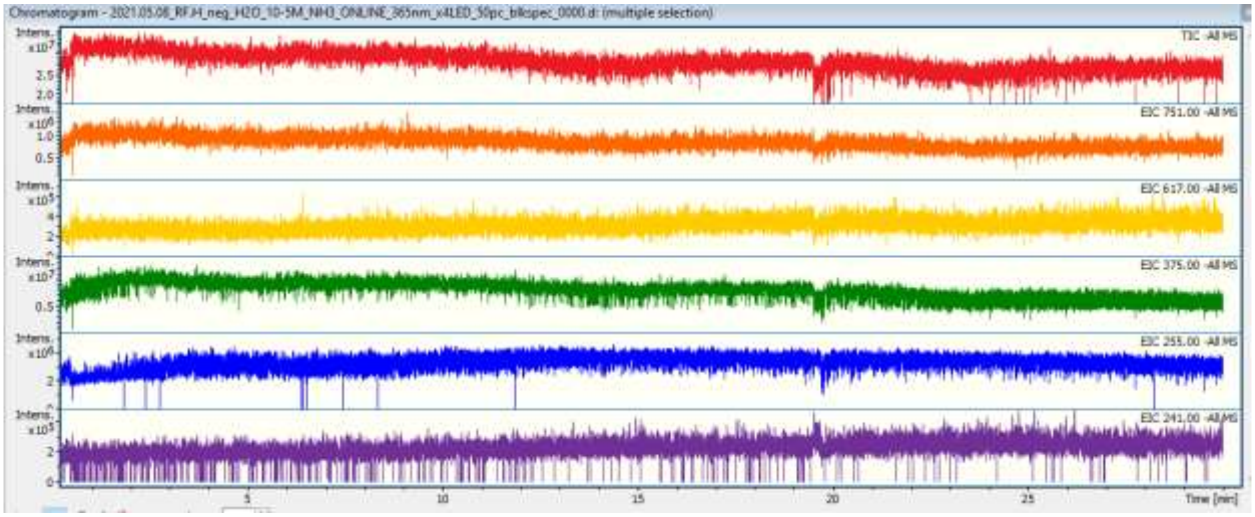

Figure S10. Representative total ion chromatogram and extracted ion chromatogram for ions at *m/z* 751, 617, 375, 255, and 241. Note that the ion intensities of the *m/z* 751, 617, 375, and 255 ions barely fluctuate relative to one another across the 0–30 min time period of irradiation. Notably the production of the major photofragment ion in the solution phase at *m/z* 241 (as identified previously in Figures 8 and 9 of the main text) is not induced when the syringe is covered, and where UV-A light is not transmitted through to the solution.

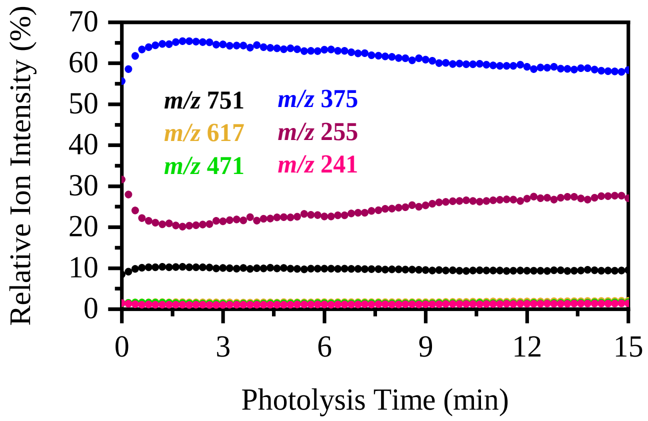

Figure S11. Relative ion intensity plot highlighting the solution-phase ions of aqueous $[RF–H]^{-}$ produced over a 30 minute interval of irradiation at 365 nm (3.4 eV) when delivered via ESI-MS in the native ion mode when the syringe is covered with a black cloth, preventing UV-A light to be transmitted into the solution for irradiation. The curved lines included with the data points are a three-point adjacent average of such data points and are extracted from an average of 3 repeat runs.

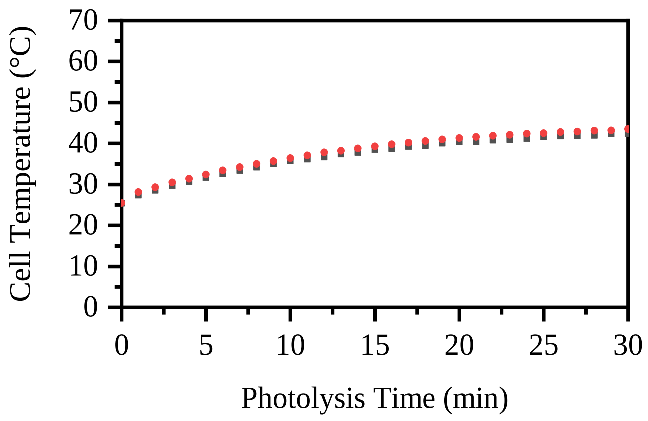

Figure S12. Plot of the data obtained *via* use of a thermocouple (Six Channel Handheld Temperature Data Logger; Omega RDXL6SD, Serial No. 003308) employed to monitor the temperature within the photolysis cell at two separate locations within the on-line photolysis cell.

References

1. Wong, N.G.K.; Rankine, C.D.; Dessent, C.E.H. Linking Electronic Relaxation Dynamics and Ionic Photofragmentation Patterns for the Deprotonated UV Filter Benzophenone-4. *J. Phys. Chem. Lett.* **2021**, *12*, 2831–2836, doi:10.1021/acs.jpclett.1c00423.

2. Berenbeim, J.A.; Wong, N.G.K.; Cockett, M.C.R.; Berden, G.; Oomens, J.; Rijs, A.M.; Dessent, C.E.H. Unravelling the Keto–Enol Tautomer Dependent Photochemistry and Degradation Pathways of the Protonated UVA Filter Avobenzone. *J. Phys. Chem. A* **2020**, *124*, 2919–2930, doi:10.1021/acs.jpca.0c01295.

3. Wong, N.G.K.; Berenbeim, J.A.; Dessent, C.E.H. Direct Observation of Photochemical Free Radical Production from the Sunscreen 2-Phenylbenzimidazole-5-Sulfonic Acid via Laser-Interfaced Mass Spectrometry. *ChemPhotoChem* **2019**, *3*, 1231–1237, doi:10.1002/cptc.201900149.

4. Matthews, E.; Dessent, C.E.H. Observation of Near-Threshold Resonances in the Flavin Chromophore Anions Alloxazine and Lumichrome. *J. Phys. Chem. Lett.* **2018**, *9*, 6124–6130, doi:10.1021/acs.jpclett.8b02529.

5. Stokes, S.T.; Bartmess, J.E.; Buonaugurio, A.; Wang, Y.; Eustis, S.N.; Bowen, K.H. Anion photoelectron spectroscopy of the linear CnH2n+1O− (n = 1–9) alkoxides. *Chem. Phys. Lett.* **2019**, *732*, 136638, doi:10.1016/j.cplett.2019.136638.
